# Epidemic Patterns of Foot and Mouth Disease in the Lao People’s Democratic Republic

**DOI:** 10.1155/tbed/8673395

**Published:** 2026-08-12

**Authors:** Ingrid Petersen, James R. Young, Syseng Khounsy, Phouvong Phommachanh, Peter Christensen, Watthana Theppangna, Tom Hughes, Stuart D. Blacksell, Michael P. Ward

**Affiliations:** ^1^ Sydney School of Veterinary Science, The University of Sydney, Camden, New South Wales, Australia, sydney.edu.au; ^2^ Mahidol Oxford Tropical Medicine Research Unit (MORU), Faculty of Tropical Medicine, Mahidol University, Bangkok, Thailand, mahidol.ac.th; ^3^ National Animal Health Laboratory, Vientiane, Laos; ^4^ Conservation Medicine, Sungai Buloh, Selangor, Malaysia; ^5^ Centre for Tropical Medicine and Global Health, Nuffield Department of Medicine, University of Oxford, Oxford, UK, ox.ac.uk

**Keywords:** FMD, hotspots, Laos, risk factors, seroprevalence, spatial analysis, surveillance

## Abstract

Smallholder livestock farming underpins food security and rural livelihoods in Lao PDR, yet the sector is threatened by endemic infectious diseases such as foot and mouth disease (FMD). Despite significant vaccination campaigns, FMD continues to circulate widely, driven by systemic challenges including limited resources, under‐reporting and unregulated animal movement. Previous studies have reported high prevalence and potential hotspots, but data remain fragmented and often exclude domestic pigs, a species that may play an important role in transmission dynamics. This study utilised abattoir‐based surveillance and spatial analysis to estimate FMD antibody seroprevalence in cattle, buffalo and pigs across Laos, identify geographic hotspots and assess animal‐level risk factors to inform more targeted and sustainable control strategies. This retrospective observational analysis used samples collected through the LACATH4 project (July 2021–May 2024). Blood samples were obtained from animals presented for slaughter and processed at the Veterinary One Health Reference Laboratory (VOHRL) in Vientiane. All sera were tested for FMD non‐structural protein (NSP) antibodies using the ID Screen FMD NSP Competition ELISA to distinguish between infection‐induced antibodies and vaccination responses. Overall, FMD antibody seroprevalence was 43% in cattle (*n* = 4587), 52% in buffalo (*n* = 3647) and 3% in pigs (*n* = 4759). Temporal patterns showed declining cattle seropositivity after 2021, increasing buffalo seropositivity in later years and a sharp rise among pigs in 2023. Female cattle and buffalo had higher odds of seropositivity than males, and pigs aged 7–12 months were more likely to test positive than younger animals. Vaccinated buffalo were more frequently seropositive, likely reflecting targeted vaccination in high‐risk areas. Spatial analysis identified species‐specific hotspots, consistent with FMD’s endemic status in Laos. As abattoir‐based samples represent a subset of the livestock population and may be biased toward specific age and sex groups, these results should be interpreted with caution. However, they provide insight into patterns of FMD exposure over time and space among animals entering slaughter channels. FMD in Laos remains endemic with species‐specific dynamics and localised hotspots, necessitating targeted surveillance and control strategies. This underscores the need for targeted vaccination and improved, population‐level surveillance strategies.

## 1. Introduction

Smallholder livestock farming is a key component of the Lao People’s Democratic Republic’s (henceforth Lao PDR or Laos) economy and food security. In 2019, Laos was ranked 87th out of 117 countries on the Global Hunger Index, indicating significant food security concerns [1]. Consequently, ~65%–73% of the total workforce lives in rural areas and are employed in the agricultural sector [1]. Livestock farming contributes around 40%–50% of the GDP, and therefore, smallholder farmers depend on livestock not only for food but also for income [2].

Infectious and transboundary diseases of livestock are a threat to food security and public health in countries like Laos [3]. Southeast Asia is a hotspot for emerging and re‐emerging zoonotic viral diseases due to rapid socio‐environmental changes [4]. The drivers of disease include urbanisation, deforestation, wildlife trade, and increased human–animal interactions [4]. However, Laos faces challenges of limited financial and human resources in controlling transboundary diseases [5].

Foot and mouth disease (FMD), caused by the FMD virus (FMDV), is a highly contagious viral disease affecting cloven‐hoofed animals [6]. FMD spreads rapidly between farms through direct contact, aerosols, and fomites [6]. There are both direct and indirect costs associated with FMD for smallholder farmers. Directly, FMD leads to immediate losses in livestock productivity, including reductions in milk yield, weight gain and fertility. These losses directly affect farmers’ incomes and the overall efficiency of livestock production systems [7]. Indirectly, FMD outbreaks disrupt trade, leading to export bans and loss of market access. The costs of implementing control measures, such as vaccination campaigns and culling infected animals, also contribute to the economic burden [7, 8]. FMD vaccination in Laos remains limited. It is not currently implemented as a comprehensive national vaccination program, relying mostly on private farmers, targeted government activities, and donor‐supported initiatives. Major support has historically come from external programs such as the Stop Transboundary Animal Diseases and Zoonoses (STANDZ) Project, which worked to strengthen animal health systems and transboundary disease control across Southeast Asia [1, 9]. Through collaboration with the SEA and China FMD Campaign (SEACFMD), ~1.6 million FMD vaccine doses were administered to large ruminants in northern Laos between 2013 and 2016 under the National Lao FMD Plan, contributing to reduced outbreak incidence and improved farmer livelihoods [1]. However, this program did not continue once the donor funding ended, and deficits persist in the region, including biosecurity, vaccine resourcing, disease surveillance, farmer engagement and disease‐response capacities [10].

FMD is considered endemic in ASEAN countries, including Cambodia, Laos, Malaysia, Myanmar, the Philippines, Thailand, and Vietnam [5]. In addition, following its reemergence in 2022, FMD is now endemic to Indonesia. Previous research has shown that the prevalence of FMD remains high in livestock populations, especially in areas without reported clinical outbreaks, implying a silent circulation of the virus [11]. FMD causes constant sporadic outbreaks in Laos and appears to be endemic despite governmental and donor project interventions [12]. There are seven recognised serotypes of FMDV: O, A, C, Asia‐1, SAT1, SAT2 and SAT3 [13]. Three different lineages have been identified in Laos: the South‐East Asian topotype, the pig‐adapted Cathay topotype, and the pan‐Asian topotype [5]. Infection with one serotype does not provide immunity to infection from another serotype [1]. When considering the persistence of post‐infection antibodies, reported durations vary between studies and host species. In cattle, non‐structural protein (NSP) antibodies following natural infection have been detected for up to 3 years, whereas experimental infections typically show persistence for around 18 months [14, 15]. In pigs, available data are limited, and few studies have monitored NSP antibody responses beyond the early post‐infection period. The existing evidence suggests that infection‐induced antibodies in pigs are relatively short‐lived, with detectable titres generally persisting for approximately 3–4 months after infection [16].

Systemic issues in Laos are hindering the control of FMD. These include a lack of sustainable funding (many programs are donor‐driven and often short‐term), limited laboratory and diagnostic capacity and ineffective disease reporting systems, which often lack coordination between local farmers and government officials [2]. Control programs, primarily based on vaccination campaigns, have been shown to be only partially effective due to challenges such as inconsistent vaccine supply, cold chain failures and limited reach into rural areas [12]. There is limited enforcement of movement restrictions on animals, and quarantine procedures are poor [11]. Unregulated livestock movements have been previously identified as a significant factor in disease spread in Laos [5] and also throughout Southeast Asia [17].

Under‐reporting from smallholder farmers is another major contributor to the low levels of FMD reporting [18]. The main reasons for under‐reporting are the fear of trade restrictions, lack of compensation, and inadequate veterinary support [2]. In one study, farmers expressed fears that their animals would be excluded from trade and lacked trust in compensation systems [1]. In addition, many farmers were unaware of the need for vaccination and stated that they lacked the resources to access veterinary care [11]. Many farmers share communal grazing and water sources, making biosecurity difficult [11]. When one animal becomes diseased in the village, infection is easily spread to the rest of the local population [19].

Previous studies have identified factors linked to hotspots in northern Laos. These include a high density of animals, seasonal animal movement, limited vaccination coverage and extensive cross‐border trade with neighbouring countries [20]. The leading causal association factor was geography, with provinces bordering other countries—such as Xieng Khouang, Luang Prabang and Houaphanh provinces—appearing to have the most hotspots [20]. Unregulated livestock movements contributed to the spread of FMD [5]. In contrast, some provinces had moderate to low rates of FMD, justifying the need for a targeted approach to disease control depending on the disease prevalence [11]. Abattoir‐based surveillance is a cost‐effective and practical approach to disease investigation [2], providing a snapshot of overall patterns of disease prevalence [2].

The aim of this study was to develop an expanded surveillance approach for FMD. By including pigs in the data collection—known to have a different cycle of FMD transmission—and incorporating spatial mapping for disease clusters, this project aimed to provide detailed insights into areas of Laos to ensure that limited government resources can be effectively allocated. Specific study objectives within this broader project include determining the current seroprevalence of FMD in buffalo, cattle and pigs in Laos; identifying spatial patterns to determine high‐risk hotspot zones for FMD in Laos; and identifying statistically significant signalment factors associated with increased risk of case classification within hotspot areas.

## 2. Material and Methods

### 2.1. Ethics Statement

The animal ethics for this study were approved by the Ministry of Agriculture and Forestry, Department of Livestock and Fisheries (DLF), Lao PDR; Approval Numbers 021‐010/National Animal Health Laboratory (NAHL) and 023‐050/DLF. The studies were conducted in accordance with local legislation and institutional requirements.

### 2.2. Study Design and Data Source

This study was designed as a retrospective observational analysis, utilising field data collected through the Laos Cambodia Thailand for One‐Health and Transboundary Disease Project (LACATH4), which operated between July 2021 and May 2024. The LACATH4 project worked in cooperation with the DLF, the NAHL, and the Mahidol Oxford Tropical Medicine Research Unit (MORU) [2, 3]. The project was funded by the United States Defence Threat Reduction Agency. Serum samples were collected from 17 provincial abattoirs across Laos as part of routine surveillance activities [21]. The methodology for sample collection and handling was adapted from the procedures described in a previous study by Gee et al. [21]. Within the last 10 days of each month, trained project staff visited each abattoir and collected serum from 10 animals of each species (cattle, buffalo and pigs) when available and sent to Veterinary One Health Reference Laboratory (VOHRL) located within the NAHL in Vientiane [21]. Approximately 10 mL of blood was collected from live animals either at the abattoir or at the farm directly before the animals were transported to the abattoir [21]. Samples were tested for FMD NSP using the ID Screen FMD NSP Competition ELISA (Innovative Diagnostics), which allows differentiation between vaccination and infection in animals [21].

### 2.3. Data Management and Statistical Analysis

Prior to the analysis, the dataset was cleaned and collated in a Microsoft Excel spreadsheet. Descriptive statistical methods were used to summarise the dataset into the number of samples per predictor category and FMD seroprevalence per category. Age categories were ≤6 months, 7–12 months, >1–2 years, >2–3 years, >3–4 years, >4–5 years, >5–6 years, >6–7 years, >7–8 years, >8–9 years, and >9 years. Body condition scoring was done using a 1−5 scale, in which 1 is emaciated and 5 is over‐conditioned. Statistical analyses were performed using Jamovi (Version 2.6, 2025) (Computer Software), available from https://www.jamovi.org.

The seroprevalence of FMD was calculated for each species (overall and also by province), with 95% confidence intervals. A *p*‐value of less than or equal to 0.05 was considered statistically significant. Chi‐squared analyses were conducted to identify statistically significant associations between categorical variables (age, breed, sampling year, sampling season, body condition, sex and vaccination status) and serostatus (positive, negative), adopting a screening significance threshold of *p* < 0.20. Variables identified as significant at the univariate level were then included in multivariate categorical logistic analyses to determine the best predictors of FMD seroprevalence, using *p* < 0.05. Model fit was assessed with the Hosmer–Lemeshow test (HL test).

### 2.4. Spatial Analysis and Hotspot Mapping

Proportional symbol maps were created to visually represent the geographic distribution of FMD seroprevalence across 17 provinces. Moran’s Index was also calculated and assesses spatial autocorrelation, with positive values indicating clustering of similar values, negative values indicating clustering of dissimilar values, and values near zero indicating spatial randomness [22]. Spatial clustering and hotspot detection were performed using SaTScan software (Version 9.6; Kulldorff M., Information Management Services, Inc., available at http://www.satscan.org). A retrospective space‐time permutation model was employed to identify significant clusters of high FMD seroprevalence. Analyses used a circular scanning window, set to a maximum of 20% of the population at risk and a temporal window of up to 20% of the total study period. This restricted window size was selected because our interest in this study was in the local dynamics of FMD occurrence. This method allowed for the detection of statistically significant spatial and temporal clusters without requiring population‐at‐risk data, facilitating the identification of potential outbreak hotspots.

## 3. Results

Table 1 presents the descriptive statistics for the study. A total of 12,993 samples were collected between 2021 and 2024, with similar sampling numbers across species (buffalo *n* = 3647; cattle *n* = 4587; pigs *n* = 4759). Most pigs were <6 months of age, whereas buffalo were more evenly distributed across age categories >3 years, and cattle were primarily 3–6 years of age. Most cattle and buffalo sampled were of native breeds, whereas pigs were predominantly of mixed native and exotic breeds. Most animals had a BCS of 3 or 4, and the vaccination status was predominantly recorded as ‘unknown’. The distribution of samples by season, sex and year appeared to be similar across species.

**Table 1:** Descriptive statistics of a survey of cattle, buffalo and pigs for foot and mouth disease antibodies, Laos, 2021–2024, in which 12,993 samples were collected and tested across three animal species (buffalo *n* = 3647; cattle *n* = 4587; pig *n* = 4759).

Variable	Category	Buffalo	Cattle	Pig	Total
Age	<6 months	1	0	3892	3893
	7−12 months	35	118	662	815
	>1−2 years	92	252	144	488
	>2−3 years	258	581	51	890
	>3−4 years	638	782	10	1430
	>4−5 years	583	870	0	1453
	>5−6 years	622	764	0	1386
	>6−7 years	449	458	0	907
	>7−8 years	567	291	0	858
	>8−9 years	180	220	0	400
	>9 years	222	251	0	473
Breed	Exotic	0	0	347	347
	Mixed	20	7	3952	3979
	Native	3627	4580	460	8667
Year	2021	88	350	309	747
	2022	1376	2138	2159	5673
	2023	1554	1616	1728	4898
	2024	629	483	563	1675
Season	Dry	2094	2552	2732	7378
	Wet	1553	2035	2027	5615
Body condition	1	0	1	0	1
	2	120	225	308	653
	3	2250	3787	1538	7575
	4	1091	535	2392	4016
	5	186	39	521	746
Sex	Male	1603	1995	1862	5460
	Female	2041	2592	2887	7519
	Unknown	3	0	10	13
Vaccination	Classical swine fever	^a^N/A	^a^N/A	396	396
	Foot and mouth disease	35	152	735	922
	No	234	179	173	586
	Unknown	3378	4256	3455	11,089

^a^Not applicable.

Tables 2 and 3 summarise estimated seroprevalences by species and by other factors investigated. Seroprevalence was ~50% in both buffalo and cattle (Table 2), whereas it was much lower in pigs (2.75%). Across age categories, seroprevalence remained relatively stable at around 50% for both buffalo and cattle. However, pigs 7–12 months of age had an increase in seroprevalence. Mixed‐breed buffalo displayed higher seroprevalence compared to native breeds, while native cattle showed higher seroprevalence than mixed‐breed cattle. Certain years showed spikes in seroprevalence, such as 2021 for cattle and 2023 for pigs, whereas 2021 showed the lowest seroprevalence overall across the 4 years studied. Buffalo vaccinated against FMDV had a higher seroprevalence than those with unknown or no vaccination history. Distribution of seroprevalence across season, sex and body condition appeared to be consistent (Table 3).

**Table 2:** Foot and mouth disease (FMD) seroprevalence by species in a survey of cattle, buffalo and pigs, Laos, 2021–2024.

Variable	Buffalo	Cattle	Pig	Total
*N* (total)	3647	4587	4759	12,993
FMD test positive	1899	1983	131	4013
FMD test negative	1748	2604	4628	8980

Seroprevalence (%) (95% CI)	52 (50%–54%)	43 (42%–45%)	3 (2%–3%)	31 (30%–32%)

**Table 3:** Foot and mouth disease (FMD) seroprevalence by species and factor in a survey of cattle, buffalo and pigs, Laos, 2021–2024.

Variable	Category	Seroprevalence (%)			
		Buffalo (%)	Cattle (%)	Pig (%)	Total (%)
Age	<6 months	100	0	2.1	2.1
	7−12 months	37.1	37.2	7.4	13
	>1−2 years	40.2	43.3	0.7	30.1
	>2−3 years	50.8	45.1	0	44.2
	>3−4 years	52	43.9	0	47.2
	>4−5 years	53.3	43.1	0	47.2
	>5−6 years	54	42.7	0	47.8
	>6−7 years	53.2	43.2	0	48.2
	>7−8 years	52	42.3	0	48.7
	>8−9 years	51.1	41.4	0	45.8
	>9 years	50.5	44.6	0	47.4
Breed	Exotic	0	0	0.6	0.6
	Mixed	70	28.6	3	3.4
	Native	52	43.3	2	44.7
Year	2021	39.0	51.4	1.3	29.3
	2022	51.7	42.4	2.1	29.3
	2023	53.9	42.4	4.1	32.5
	2024	50.2	43.9	1.8	32.1
Season	Dry	51.3	43.9	2.1	30.6
	Wet	53.1	42.4	3.6	31.3
Body condition	1	0	0	0	0
	2	50.8	41.3	3.9	25.4
	3	52.1	43.8	2.6	37.9
	4	52.2	^b^40.8	3	21.4
	5	51.6	38.5	1.5	16
Sex	Male	50.8	39.6	2.8	30.3
	Female	53.1	46	2.7	31.3
	Unknown	66.7	0	0	15.4
Vaccination	Classical swine fever	^a^N/A	^a^N/A	1.8	1.8
	Foot and mouth disease	68.6	44.1	3.4	12.6
	No	45.7	39.7	0	30.4
	Unknown	52.3	43.4	2.9	33.5

^a^Listed as vaccination only for pigs.

^b^Two estimates were included that were reported as 3.5 body condition and were merged into category 4.

Univariate chi‐squared tests identified variables with an association (*p* ≤ 0.20) with serostatus for inclusion in the logistic regression analyses. For buffalo, the breed, vaccination status, and year of sample collection met this criterion. For cattle, the breed, sex and year of sample collection were included. For pigs, body condition, age and year of sample collection were included.

Given the results of univariable analyses, in which year of sampling appeared to be a strong predictor of FMD serostatus, multivariable logistic regression models were fit for each species adjusted for the sampling year. In all cases, only a single variable was retained in each species‐specific model (Table 4). For buffalo, vaccination status was significantly associated with FMD positivity: buffalo with a ‘no’ or ‘unknown’ vaccination history had 65% and 44% lower odds of testing positive, respectively, compared to vaccinated buffalo. For cattle, sex was significantly associated with FMD test seropositivity, with males 23% less likely to test positive than females. Pigs 7–12 months of age were significantly 3.91 times more likely to test positive than pigs <6 months of age. Buffalo and cattle multivariable models were an adequate fit to the data (Hosmer and Lemeshow *p* = 0.992 and 0.665, respectively); however, the pig model was not (Hosmer and Lemeshow *p* = 0.013).

**Table 4:** Factors associated with foot and mouth disease (FMD) seropositivity in a survey of cattle, buffalo and pigs, Laos, 2021–2024.

Species	Factor	Category	Estimate	SE	OR	95% CI
Buffalo	Vaccine	No vs. FMDV	−1.059	0.391	0.35	0.16, 0.75
		Unknown vs. FMDV	−0.794	0.370	0.45	0.22, 0.93
	Year	2023 vs. 2021	0.60	0.23	1.82	1.17, 2.84
Cattle	Sex	Male vs. female	−0.262	0.0606	0.77	0.68, 0.87
	Year	2022 vs. 2021	−0.36	0.12	0.70	0.55, 0.88
		2023 vs. 2021	−0.37	0.12	0.68	0.55, 0.87
Pigs	Age	7−12 months vs. ≤6 months	1.365	0.198	3.91	2.66, 5.77
		>1−4 years vs. ≤6 months	−1.663	1.011	0.19	0.03, 1.38
	Year	2023 vs. 2021	1.52	0.53	4.50	1.60, 12.8

*Note:* Reference category for each comparison is listed on the right of the figure.

**Figure 1:**
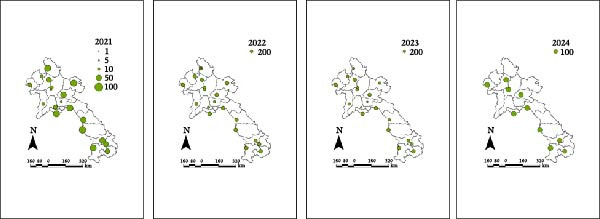
Spatial distribution of sample collection sites in a survey of foot and mouth disease (FMD) in Laos from 2021 to 2024, mapped per sample collection year. Green circles represent the number of samples collected per site, with circle size proportional to sampling intensity (see legends for each panel). Provincial boundaries, north arrow and scale bar (km) are provided for reference. Circle size scales differ between years to reflect variation in sample numbers.

**Figure 2:**
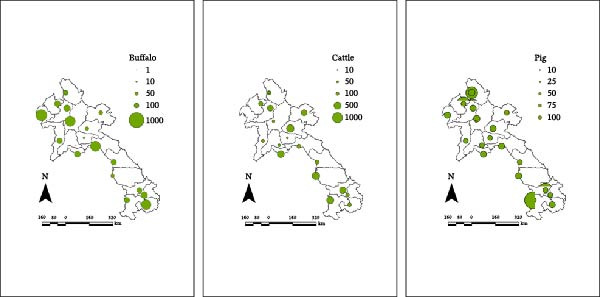
Spatial distribution of FMD sample collection sites across Laos per species. Green circles indicate the number of samples collected per site, with circle size proportional to sample count. Provincial boundaries, north arrow and scale bar (km) are shown for reference.

**Figure 3:**
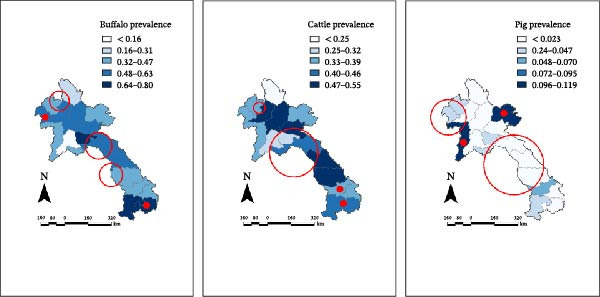
Estimated foot and mouth disease (FMD) seroprevalence by species (buffalo, cattle and pigs) in a survey in Laos, 2021–2024. Darker shading representing higher seroprevalence (see colour scale). Clusters are shown by red open circles proportional to cluster radius (km). Closed red circles indicate a cluster detected at only one province location (centroid), hence a radius of 0 km. Provincial boundaries, north arrow and scale bar (km) are shown for reference.

Figure 1 shows the distribution of FMD samples collected across Laos during the study period per year. Samples were obtained from all provinces, although the collection intensity varied between regions and years. The largest number of samples was collected in 2021, while 2024 had the lowest. When viewing sample collection by species, there was a relatively homogeneous spread across Laos (Figure 2). Provincial estimates of seroprevalence ranged from 16% to 80% in buffalo, 25% to 55% in cattle and 0.02% to 2% in pigs. In buffalo, while seroprevalence was moderately high across the entire country, two provinces in the west and south of Laos recorded the highest disease seroprevalence (Figure 3). In cattle, seroprevalence was highest in the central provinces (Figure 3). In pigs, seroprevalence was relatively low overall compared to cattle and buffalo; however, the northwest and east provinces had the highest seroprevalence (Figure 3). Moran’s Index values for seroprevalence were −0.16 for buffalo, 0.03 for cattle and −0.11 for pigs (*p* = 0.62, 0.69 and 0.81, respectively), suggesting no overall global clustering of provincial seroprevalence estimates.

Spatial clusters were distributed across different regions for all animal species, with distances ranging from local points (0 km radius) to over 252 km, indicating multiple independent foci of elevated‐risk areas. Five significant clusters were identified in buffalo (Table 5), with Clusters 2 and 3 showing the highest relative risk (O/E = 1.86, *p* < 0.001) and Cluster 4 contributing the largest case burden (220 cases). In cattle, four clusters were detected; Cluster 1 accounted for the greatest number of cases (791 observed), while Cluster 3 showed the highest relative risk (O/E = 2.23, *p* < 0.001). In pigs, four clusters were also observed, with Cluster 1 representing both the greatest case burden (19 cases) and the highest relative risk (O/E = 34.51, *p* < 0.001). The temporal length of clusters ranged from 1 to 654 days, tending to be shorter for pigs (average 59 days) than buffalo (197 days) or cattle (271 days) and occurred throughout different times of the year.

**Table 5:** Spatial–temporal scan of statistically significant foot and mouth disease clusters for buffalo, cattle and pigs in Laos, 2021–2024.

Species	Parameters	Cluster 1	Cluster 2	Cluster 3	Cluster 4	Cluster 5
Buffalo	Coordinates	14.8178 N, 106.8179 E, 0 km	16.5749 N, 104.7668 E, 110 km	20.9671 N, 101.4056 E, 90 km	20.2628 N, 100.4340 E, 0 km	18.3763 N, 103.7284 E, 132 km
	Time frame	20/2/2023−20/11/2023	17/9/2023−19/11/2023	25/10/2022−29/11/2023	20/6/2022−20/11/2022	26/6/2022−28/9/2022
	Number of cases	135	30	29	220	56
	Expected cases	81	16	16	175	36
	Observed/expected	1.67	1.86	1.86	1.26	1.56
	*p* value	<0.001	<0.001	<0.001	0.0042	0.0068
Cattle	Coordinates	18.3763 N, 03.7284 E, 229 km	15.7014 N, 106.4044 E, 0 km	15.0914 N, 105.6412 E 0 km	20.9671 N, 101.4057 E, 68 km	…
	Time frame	2/11/2021−17/8/2023	21/11/2023−27/1/2024	21/1/2023−21/2/2023	22/6/2023−21/5/2024	…
	Number of cases	791	36	26	89	…
	Expected cases	670	17	12	56	…
	Observed/expected	1.18	2.14	2.23	1.58	…
	*p* value	<0.001	<0.001	<0.001	<0.001	…
Pigs	Coordinates	20.4171 N, 104.0481 E, 0 km	17.4021 N, 104.8378 E, 252 km	18.7021 N, 101.5393 E, 0 km	20.2628 N, 100.4340 E, 127 km	…
	Time frame	23/5/2023−22/6/2023	26/7/2022−10/8/2022	16/12/2022−25/6/2023	26/9/2022−26/9/2022	…
	Number of cases	19	13	19	7	…
	Expected cases	0.6	0.6	1.9	0.3	…
	Observed/expected	35	24	10	25	…
	*p* value	<0.001	<0.001	<0.001	<0.001	,,,

*Note:* Cluster coordinates represent the geographic centre of each statistically significant (*p* < 0.05) cluster, with distances given as cluster radius (km). The time frame indicates the period during which clustering was detected. Number of cases refers to the observed count within the cluster detected, and expected cases represent the number predicted under the null hypothesis of random distribution. The observed/expected ratio indicates the relative risk within the cluster. *p* values were obtained using Monte Carlo simulation.

## 4. Discussion

This study represents one of the most comprehensive investigations of FMD seroprevalence in Laos to date, capturing patterns across all provinces over a 4‐year period. By integrating systematic abattoir‐based antibody testing in three major livestock species, the survey provides a detailed overview of national FMD dynamics. The findings reveal clear species‐specific differences in seroprevalence and highlight distinct geographical hotspots of infection, offering new insights into the epidemiology of FMD in Laos.

The seroprevalence data from our study revealed notably low seropositivity in pigs (~3%), in contrast to significantly higher seropositivity observed in cattle (43%) and buffalo (52%). These findings are consistent with those reported by Siengsanan‐Lamont et al. [23], who documented seroprevalences of 45% in cattle, 35% in buffalo and 1.3% in pigs. Notably, our buffalo seroprevalence was higher than previously reported, which might be attributed to a larger sample size and a greater representation of buffalo in our sample. An alternative explanation, as demonstrated in Kenya by Omondi et al. [24], supports an argument that buffalo play a more substantial role in the transmission and spread of FMD than previously assumed. In addition to this, Moran’s index for seroprevalence for each species (−0.16 for buffalo, 0.03 for cattle and −0.11 for pigs) supports the conclusion that overall FMD is endemic in Laos despite the occurrence of local hotspots.

Yearly seroprevalence trends varied between sampled species: the highest seroprevalences were estimated for buffalo during 2022−2024, whereas this was a period of lower seroprevalences for cattle and pig seroprevalences. This latter trend might be linked to shifts in commercial pig farming practices in Laos, where a notable expansion occurred post‐2022, followed by a sharp (~30%) decline in the pig population [25]. Such abrupt demographic changes could have led to increased animal movement or rapid depopulation efforts, potentially facilitating the spread of FMD. For buffalo, there was a more gradual rise in seropositivity across the sampled years. Given that buffalo in Laos serve multiple roles—including ploughing and meat production—it is plausible that the observed increase reflects increased buffalo utilisation and perhaps increased cohabitation with other FMD carrier species. The trend in cattle was particularly noteworthy, characterised by a consistent decline in FMD seropositivity over the sampled years. Several factors may account for this reduction, including enhanced vaccination coverage, improved farm‐level biosecurity, decreased stock movement or reduced herd exposure due to the dominance of a single FMD serotype [21]. The relatively short duration of immunity following FMD infection and vaccination likely contributes to the observed decline in seroprevalence over time. Protective antibody levels can wane within a few months, necessitating booster vaccination every 3–6 months to sustain herd immunity in endemic regions. In such cases, once animals are exposed and develop antibodies, subsequent serological responses may be diminished, resulting in lower detection rates over time.

Analysis of vaccination status revealed a counterintuitive finding: vaccinated animals exhibited a higher FMD seroprevalence than those that were unvaccinated or had an unknown vaccination status. Further analysis revealed that buffalo with ‘no’ or ‘unknown’ vaccination history had 65% and 44% lower odds, respectively, of testing positive for FMD, compared to vaccinated buffalo. The diagnostic assay employed in this study targeted NSPs of FMDV, enabling differentiation between vaccinated and infected animals. Therefore, positive results reflect true infection rather than vaccine‐induced seropositivity. Although no prior data from Laos exist regarding vaccination and FMD seroprevalence, studies from other countries generally report an inverse relationship between vaccination coverage and disease seroprevalence. One plausible explanation for our findings is that vaccination efforts in Laos are concentrated in outbreak‐prone regions; hence, vaccinated animals are more likely to come from areas of FMD outbreaks, making them inherently at higher risk of exposure. Additionally, current vaccines do not provide coverage against all circulating FMDV serotypes, leaving animals vulnerable to infection by heterologous strains. Another possible explanation is that vaccine purity could be creating true false‐positive test reactions. This is supported in the literature where NSP contamination in vaccines can create false positives and it is likely that poor manufacturing or storage of the vaccinations, such as bad batches (i.e., expired vaccine, cold‐chain failure), lead to vaccinated animals not being protected against FMD [26].

Age‐stratified analysis indicated that pigs 7–12 months of age exhibited the highest seropositivity, whereas cattle and buffalo showed more evenly distributed seroprevalence across age groups. This age pattern in pigs likely reflects an earlier age of marketing and slaughter compared to that in cattle and buffalo. Pigs are typically slaughtered at 7–12 months of age, suggesting that younger pigs may benefit from residual maternal antibodies or are housed in more protected environments, thereby limiting their exposure to FMDV [26]. Lower seroprevalence in pigs has been reported previously [27].

In cattle, a sex‐based predisposition was observed: males were 23% less likely to test positive for FMD than females. This disparity might be explained by production practices in Laos, where males are typically slaughtered at a younger age, reducing their cumulative exposure to FMD. In contrast, females are retained longer for reproductive purposes, with the average age at first calving reported to be 2.5–3 years [28].

Spatial analysis revealed that FMD provincial seroprevalence ranged from 16% to 80% in buffalo, 25% to 55% in cattle and 0.02% to 2% in pigs. A total of four hotspots were identified for cattle and pigs and five for buffalo. These hotspots varied considerably in geographic extent and case density. This spatial pattern reinforces the conclusion that FMD remains an endemic disease in Laos with local hotspots; this shows the value of testing for global clustering (Moran’s I) as well as searching for local hotspots of disease to understand the complex epidemiology of diseases such as FMD in developing countries. Our findings are consistent with previous literature, which has primarily focused on northern Laos for FMD hotspot mapping. While our results confirm similar seroprevalence and hotspot locations, they also demonstrate widespread FMD distribution across all provinces, with concentrated hotspots in the Xayabury and Hauphanh regions [3, 29]. Notably, our data indicate higher seroprevalence rates in Champasack and Attapeu for buffalo and cattle than previously reported [3, 29]. Pig hotspot data also aligned with earlier findings in the Xayabury and Hauphanh regions; however, prior studies aggregated data across species, limiting direct comparison [29]. Potential drivers of these hotspot areas include trade routes, animal movement patterns, environmental conditions and cultural practices. Among these, trade and animal movement appear to be the most influential. Previous studies have shown that while most livestock movement by smallholder farms occurs locally (within 6 km), there is also targeted movement to specific provinces for sale or slaughter, notably Champasack, Savannakhet and Xiangkhouang [30]. Additional village‐level factors may also contribute to FMD hotspot formation, such as proximity to international borders, the volume of large ruminant flow through provinces and the distance from villages to official roads or national border crossings [1]. These non‐animal factors warrant further investigation to identify critical control points within the production and distribution chain, thereby informing more effective FMD mitigation strategies.

This study has several limitations. Vaccination status was difficult to correlate with seroprevalence as it was unknown for most animals, largely due to smallholder production systems with limited traceability. This is a critical data gap that needs to be addressed to enable more detailed insights to be generated on FMD control in the smallholder sector. Other recorded data, such as age and breed, might also be unreliable because of inconsistent traceability and reliance on farmers’ records. Results generated from the pig logistic regression model need to be interpreted cautiously, given that this model had an inadequate fit to the data. The very low seroprevalence we found in pig samples makes interpretation of risk factor analysis problematic. Abattoir‐based surveillance, while effective for obtaining large sample sizes representative of local populations, can be biased toward livestock that are unwell or culled rather than reflecting the general healthy population. Conversely, for diseases that cause mortality, an abattoir‐based survey can underestimate disease seroprevalence. In addition, the spatial analysis assumed that animals slaughtered at each abattoir were born and raised in the corresponding province. We considered this to be a reasonable assumption for the livestock population in Laos.

## 5. Conclusion

This study demonstrates that FMD remains endemic in Laos, with clear species‐specific differences, temporal variability and geographically distinct hotspots. These findings highlight the need to strengthen surveillance systems that integrate abattoir monitoring with active field sampling and vaccination records to better capture real‐time infection dynamics. FMD control efforts in Laos should prioritise expanding longitudinal, population‐based surveillance to complement abattoir data; implementing risk‐based biosecurity and vaccination programs that target identified hotspots and high‐mobility areas; improving traceability of livestock movements between provinces and across borders; and enhancing farmer engagement and reporting through community‐based animal health networks. Together, these steps would support a more coordinated, data‐driven approach to FMD control—improving early detection, reducing transmission and strengthening the resilience of the smallholder livestock sector. Future research should investigate the drivers of the identified hotspots, including trade routes and environmental factors, to inform more effective control strategies. Other potential research could involve transboundary trade routes and seroprevalence studies in neighbouring countries to better understand regional FMD prevalence.

## Funding

The project or effort depicted was or is sponsored by the Department of Defense, Defense Threat Reduction Agency. The content of the information does not necessarily reflect the position or the policy of the federal government, and no official endorsement should be inferred (Grant HDTRA1‐08‐D‐0007). This research was funded in whole or in part by the Wellcome Trust (Grant 220211/Z/20/Z) of the United Kingdom. For Open Access, the author has applied a CC‐BY public copyright licence to any author accepted manuscript version arising from this submission. This research was (in part) funded by the Sydney School of Veterinary Science Research & Enquiry Unit of Study 2025 fund. Michael P. Ward is the recipient of an Australian Research Council Australian Laureate Fellowship (Grant FL240100037) funded by the Australian Government.

## Conflicts of Interest

The authors declare no conflicts of interest.

## Data Availability

The data that support the findings of this study are available from the corresponding author upon reasonable request.
